# Pneumatic retinopexy in the real world: A critical reappraisal of the PIVOT trial

**DOI:** 10.1038/s41433-025-04143-z

**Published:** 2025-12-03

**Authors:** Hashem Abu Serhan, Asmaa A. Youssif, Ameen Alkhateeb

**Affiliations:** 1 Department of Ophthalmology, Hamad Medical Corporation, Doha, Qatar; 2 Feinberg School of Medicine, Northwestern University, Chicago, IL USA

**Keywords:** Retinal diseases, Vitreous detachment, Education

In their landmark the Pneumatic Retinopexy versus Vitrectomy for the Management of Primary Rhegmatogenous Retinal Detachment Outcomes Randomized Trial (PIVOT) trial, Hillier et al. [1] challenged conventional surgical practice by demonstrating that pneumatic retinopexy (PnR) can achieve superior visual outcomes compared to pars plana vitrectomy (PPV), positioning PnR as a compelling first-line option for selected cases of primary rhegmatogenous retinal detachment (RRD). The authors reported a primary anatomic success rate of 80.8% for PnR versus 93.2% for PPV, along with superior visual acuity (VA) and patient-reported outcomes in the PnR group. While PIVOT provides valuable evidence under appropriately controlled conditions, a broader review of real-world data and recent meta-analyses indicates that these results likely overestimate the generalisable efficacy of PnR.

The PIVOT trial’s most significant limitation lies in its definition of primary anatomic success. The reported 80.8% success rate for PnR includes seven cases that required gas reinjection, a distinct second procedure [1]. Adhering to the standard definition of Single-Operation Success (SOS) used in large registries, the recalculated success rate for PnR in PIVOT falls to approximately 71.8%. Thus, nearly one in four PnR patients (28.2%) in PIVOT required a second intervention compared with only 6.8% in the PPV group. This disparity has direct implications for patient burden, anxiety, recovery, and healthcare costs factors not adequately represented in the trial’s conclusions.

The trial was conducted under ideal conditions i.e., highly experienced surgeons, narrow inclusion criteria (e.g., superior breaks, limited detachment extent), and high-resource settings. Although these parameters ensure internal validity, they do not reflect the complexity of real-world retinal practice. A nationwide analysis from the IRIS® Registry by Yannuzzi et al. [2], including 9,659 eyes, reported an SOS rate of 68.5% for PnR. Other series corroborate these lower rates: Modi et al. [3] reported 63%, and Castanos et al. [4] observed 46.4%, rising to 51.9% after excluding temporising cases. When the corrected PIVOT rate is considered, real-world studies consistently converge around 60–70% SOS, consistent with the lower end of the range (45–80%) in systematic reviews [5, 6] (Table 1).

**Table 1:** Summary of PnR primary success across studies.

Study	Data Source	Sample Size (Eyes)	PnR Primary Success (%)
PIVOT (Hillier et al., 2019) [1]	Randomised Controlled Trial	88	80.8
PIVOT (Recalculated SOS) [1]	Randomised Controlled Trial	78	71.8
IRIS Registry (Yannuzzi et al., 2021) [2]	Real-World Registry	9659	68.5
Modi et al., 2014 [3]	Academic Centre Review	137	63
Castanos et al., 2024 [4]	Institutional Series	282	46.4

^*^ *PnR* Pneumatic Retinopexy, *SOS* Single-Operation Success.

PIVOT’s claim of superior visual outcomes with PnR should be interpreted cautiously due to a major confounding factor—cataract progression. The study reported that 65% of phakic patients in the PPV arm required cataract surgery within 12 months, compared with 16% in the PnR group (*P* < 0.001) [1]. PPV is known to accelerate cataract formation through increased intraocular oxygen exposure and light toxicity [7]. Consequently, the apparent visual benefit of PnR at 12 months largely reflects PPV’s lens-related complications, not an intrinsic functional superiority of the PnR technique. The meta-analysis by Roshanshad et al. [8] reinforces this interpretation: While PnR patients had slightly better final VA, the magnitude of improvement from baseline was greater in PPV. When controlling for lens status (e.g., pseudophakic subgroups or post-cataract eyes), the visual advantage of PnR diminishes or disappears entirely.

The PIVOT trial’s restrictive inclusion criteria excluded many common RRD scenarios, particularly pseudophakic detachments. Pseudophakic retinal breaks are often small, anterior, and multiple, challenging to visualise and manage effectively with an office-based procedure like PnR [5, 9]. Both the IRIS Registry [2] and Zaidi et al. [9] reported lower success in pseudophakic eyes (44%) compared with phakic eyes (58%). The meta-analysis by Roshanshad et al. [8] further demonstrated that PPV’s advantage was most pronounced in studies with fewer phakic eyes, highlighting lens status as a key effect modifier. Moreover, uncontrolled or advanced glaucoma, a condition frequently encountered in practice, is a recognised contraindication to PnR due to postoperative intraocular pressure spikes [5, 10]. These factors limit the applicability of PnR to a much smaller, highly selected subset of patients than PIVOT implies.

The PIVOT study and subsequent ALIGN study by Francisconi et al. [11] showed that PnR resulted in significantly less retinal displacement (14.7%) than PPV (50.7%), potentially explaining the reduced metamorphopsia and better patient-reported quality of life. However, this functional advantage should be weighed against its substantially higher primary failure rate. For the roughly one-third of real-world PnR patients who require reoperation, the psychological, financial, and visual impact of recurrence likely offsets any early quality-of-life benefit. The clinical relevance of less retinal displacement should therefore be considered in context of its trade-offs in anatomic reliability.

In summary, PnR remains a valuable, minimally invasive, and cost-effective option for a well-selected subset of patients—those with superior, limited detachments and reliable posturing. However, real-world data and meta-analytic evidence strongly caution against generalising the PIVOT results. The trial’s inclusion of gas reinjection as primary success, its idealised conditions, and the influence of cataract confounding collectively overstate PnR’s true efficacy. In routine practice—particularly for pseudophakic, inferior, or complex detachments—PPV remains the more reliable and broadly applicable approach. A balanced, evidence-based strategy that integrates randomised trial data with real-world outcomes and patient-specific factors is essential. The PIVOT trial should thus be viewed as defining the upper limit of PnR’s potential in ideal circumstances, not a new standard for its general application.

## Data Availability

All the data used in this study are available within the article.
